# Integrated Molecular, Genomic, and Clinical Characterization of Pediatric and Adolescent Translocation Renal Cell Carcinoma: A Report from the Children’s Oncology Group

**DOI:** 10.3390/biomedicines14050955

**Published:** 2026-04-22

**Authors:** Alissa Groenendijk, Bruce J. Aronow, Nicholas Cost, Mariana Cajaiba, Lindsay A. Renfro, Elizabeth J. Perlman, Lisa Dyer, Teresa A. Smolarek, Elizabeth A. Mullen, Sameed Pervaiz, Somak Roy, Phillip J. Dexheimer, Peixin Lu, Peter F. Ehrlich, M. M. van den Heuvel-Eibrink, Jeffrey S. Dome, James I. Geller, on behalf of the COG Renal Tumor Committee

**Affiliations:** 1 Princess Máxima Center for Pediatric Oncology, 358 CS Utrecht, The Netherlands; 2 Division of Biomedical Informatics, Cincinnati Children’s Hospital Medical Center, Cincinnati, OH 45229, USA; 3 Department of Surgery, Division of Urology, University of Colorado School of Medicine and the Children’s Hospital Colorado, Aurora, CO 80045, USA; 4 Department of Pathology, University of Michigan Medical Center, Ann Arbor, MI 48109, USA; 5 Division of Biostatistics, University of Southern California, and Children’s Oncology Group, Los Angeles, CA 90089, USA; 6 Department of Pathology and Laboratory Medicine, Ann & Robert H. Lurie Children’s Hospital of Chicago, Robert H. Lurie Cancer Center, Northwestern University, Chicago, IL 60611, USA; 7 GeneDx, Gaithersburg, MD 20877, USA; 8 Department of Pediatrics, University of Cincinnati, Cincinnati, OH 45229, USA; 9 Department of Pediatric Oncology, Children’s Hospital Boston, Dana-Farber Cancer Institute, Boston, MA 02215, USA; 10 Division of Pathology, Cincinnati Children’s Hospital Medical Center, University of Cincinnati, Cincinnati, OH 45229, USA; 11 Department of Surgery, Section of Pediatric Surgery, University of Michigan, Ann Arbor, MI 48109, USA; 12 Child Health, Wilhelmina Children’s Hospital, University Medical Center Utrecht, 358 CS Utrecht, The Netherlands; 13 Division of Pediatric Oncology, Children’s National Medical Center, Washington, DC 20010, USA; 14 Division of Hematology/Oncology, Peckham Center for Cancer and Blood Disorders, Rady Children’s Hospital, San Diego, CA 92123, USA; 15 Department of Pediatrics, University of California, La Jolla, CA 92092, USA

**Keywords:** pediatric, renal cell carcinoma, translocation type, molecular biology

## Abstract

**Background**: Translocation morphology renal cell carcinoma (tRCC) accounts for nearly half of all pediatric RCC cases. Biological study AREN14B4-Q aimed to characterize the molecular landscape of tRCC using samples acquired from patients enrolled in the Children’s Oncology Group Risk Classification and Biobanking study AREN03B2. **Methods**: From 2006 to 2014, patients <30 yr old with renal tumors were prospectively enrolled in AREN03B2, a Central IRB-approved biobanking study. All pediatric RCC cases underwent a detailed central pathology review and molecular diagnostics to accurately classify RCC subtypes. Samples with confirmed tRCC and appropriate informed consent were identified with adequate tissue for RNA and DNA extraction, along with germline DNA, for whole-genome sequencing (WGS), RNA sequencing, and DNA methylation analyses. **Results**: From 41 patients, high-quality samples allowed for 18 tumors and non-tumor DNA to be analyzed via WGS, 19 via DNA methylation, and 36 RNA samples via transcriptome sequencing. Consistent with and extending clinical cytogenetic findings, WGS and fusion transcript analyses confirmed very few additional mutations beyond the tRCC translocation. No recurrent genomic copy number gains/losses were found. RNA and WGS analyses enabled sub-classification of tRCC, closely aligning with the different TFE3 fusion partners. DNA methylation analyses demonstrated less tRCC sub-stratification compared with RNA analyses. Pathways activated in tRCC were involved in epithelial differentiation, extracellular matrix organization, apoptosis, immune regulation, signal transduction, and angiogenesis. **Conclusions**: Arrested epithelial differentiation is the overarching driver in tRCC and is strongly correlated with the specific subclasses of fusion transcript generated by the genetic translocation TFE fusion partner. Negative regulation of apoptosis, increased M2 macrophage expression, and enhanced angiogenesis also appear to be functional features of tRCCs, as are increased expression of matrix metalloproteinases, PI3K-AKT/mTOR/MAPK signaling, and mitochondrial metabolism, highlighting potential therapeutic options beyond direct targeting of the oncogenic driver fusions.

## 1. Introduction

Approximately 82,000 new cases of renal cell carcinoma (RCC) are diagnosed each year in the United States, resulting in almost 15,000 deaths [1,2]. In the pediatric population, RCC is the second most common form of renal malignancy, accounting for 3–6% of renal cancers in children and adolescents. Depending on upper-age cutoff (ranging from 18 to 21 years), the median age at diagnosis of pediatric RCC (pRCC) is between 9 and 12 years, with equal prevalence in both males and females before the age of 15 and higher prevalence in females among adolescents older than 15 [1,3]. RCC is a heterogeneous disease based on differences in morphology, genetic alterations, and clinical behavior. Recent studies suggest that pRCC is different from adult RCC [1,4,5], clinically manifested by the three-fold improved survival in pRCC patients with N + M0 disease when compared with similarly staged adult RCC patients [6], suggesting biological differences. More recently, translocation morphology RCC (tRCCs) was found to account for 48% of pRCCs [1,7] and classically results from gene fusions between the transcription factor E3 (TFE3) gene located on chromosome Xp11.2 with 1 of over 20 various fusion partners reported to date [8,9]. In addition to TFE3, fusion may also involve the transcription factor B (TFEB) gene on chromosome 6p21, with or without a concomitant TFEB amplification [10].

tRCC biological heterogeneity thus far remains poorly characterized and is, in fact, often challenging to subclassify [11,12]. Current diagnostic methods include histologic appearance and TFE3/TFEB status through immunohistochemistry [13,14,15,16]. However, the sensitivity and specificity of these approaches is limited. Histological appearance is both non-specific by itself and variable with respect to relative proportions and patterns of papillary structures, clear cells, and psammoma bodies between cases. TFE3 immunostaining can give false-positive and false-negative results. TFE3 break-apart fluorescence in situ hybridization (FISH) analysis is a gold standard when present but unclear regarding sensitivity for non-canonical translocations. Additional RNA sequencing or RT-PCR can assist in the diagnosis but also mostly relies on canonical translocation models [1,17].

Loss of heterozygosity (LOH) and DNA copy number abnormalities with prognostic value have been described in adult Clear Cell RCC [18]. Similarly, one study demonstrated that in 91% of morphologically challenging or complex adult RCC cases, the virtual karyotype generated by single-nucleotide polymorphism (SNP) microarray analyses unambiguously detected the presence or absence of chromosomal aberrations characteristic of one of the common subtypes of renal epithelial tumors, while immunohistochemistry and FISH demonstrated relatively less utility [19,20].

Small studies (including only a few pediatric tRCC cases) have preliminarily demonstrated tRCC biological heterogeneity, showing differences in copy number alterations per fusion subtype, as well as molecular clusters (transcriptome based) with divergent pathway signatures, associated with fusion partners [1,9,21,22,23]. Investigation into the molecular and biological drivers of pediatric tRCC, specifically, holds promise to improve the diagnostics and sub-classification of pediatric tRCC and, importantly, to identify targets amenable to therapeutic intervention, enabling more tailored therapy for infants, children, adolescents, and young adults with advanced tRCC.

## 2. Materials and Methods

### 2.1. Study Population

The COG AREN03B2 study (NCT00898365) enrolled 4521 eligible patients on or before 31 December 2014, of whom 75 had tRCC per central review (including histology, TFE immunohistochemistry, and secondary genetic analyses), as previously described [1], and central review staging using the TNM staging system. All participants were consented at participating institutions with AREN03B2 approved by their local Institutional Review Board (IRB) and/or via the Central IRB. Cajaiba et al. previously described tRCC patients enrolled in AREN03B2 [1]. The patient cohorts described in this current study and described by Cajaiba et al. do not fully overlap, as our work was performed on the cases for which we had adequate tissue for deeper biology, including WGS, methylation, and RNA profiles. For the comparison between our tRCC cases to adult RCCs, all patients from the Cancer Genome Atlas (TCGA) kidney projects were included in the analysis.

### 2.2. Fish

Fluorescence in situ hybridization (FISH) analysis was used to detect TFE3 and TFEB gene rearrangements, with TFE3 probes designed, validated, and used clinically to detect TFE-positive RCC at the Cincinnati Children’s Hospital Cytogenetic Laboratory. These probes include the TFE3 break-apart rearrangement probe (Cytocell, Oxford, UK), the PRCC::TFE3 [t(X;1)(p11.2;q21.2)] (Cytocell) and ASPL::TFE3 [t(X;17)(p11.2;q25.3)] (Empire Genomics, Buffalo, NY, USA) dual-color fusion probe sets, and the TFEB (6q21) break-apart rearrangement probe (Empire Genomics). Hybridization signals were scored in 250 cells by two independent technologists in areas with high tumor cell content, as previously described [1].

### 2.3. DNA and RNA Extractions

DNA from matched normal kidney and tRCC samples was extracted from FFPE material using the QIAamp DNA FFPE Tissue Kit (Qiagen, Germantown, MD, USA). DNA extraction from blood was performed using the DNeasy Blood and Tissue Kit (Qiagen). Total RNA was extracted from FFPE tissue samples with the Qiagen Rneasy kit (Qiagen) without DNase treatment. All extractions were performed as per the manufacturer’s protocol.

### 2.4. Whole-Genome Sequencing

Double-stranded DNA, isolated from normal (blood or kidney) and tumor samples (1 µg as determined by Invitrogen Qubit measurement (ThermoFisher, Waltham, MA, USA), was sheared by sonication to an average size of 200 bp using a Covaris S2 instrument (Covaris LLC, Woburn, MA, USA). Library construction was performed on an IntegenX Apollo324 (IntegenX Inc, San Diego, CA, USA). Following 9 cycles of PCR amplification using the Clontech Advantage II kit (Takara Bio USA, San Jose, CA, USA), 1 µg of genomic library was recovered for exome enrichment using the NimbleGen EZ Exome V2 kit (Roche NimbleGen Inc, Madison, WI, USA). The library was sequenced on an Illumina HiSeq2500, generating 150 bp paired-end reads with approximately 30 million reads per sample (Illumina, San Diego, CA, USA). The reads were aligned to the GRCh37 reference genome with BWA-MEM (v0.7.15) [24]. Tumor and normal samples had mean coverages of 24× and 28×, respectively, with insert sizes averaging 335 bp for tumor and 351 bp for normal. Duplicate reads, qc-failed reads, and reads with >5 bp clipping or <60 bp length were excluded, as were discordant pairs with insert sizes outside 234–514 bp (tumor) or 239–619 bp (normal). Subsequently, variant calling was performed using GATK (version 3) [25], and SvABA with the dbSNP database was used for structural variant detection [26]. Both methods compared normal and tumor DNA. The variants were annotated with VEP and dbNSFP [27,28]. The genomic alterations were visualized with OncoPrint [29]. Recurrent variants were considered missense/in-frame and/or loss-of-function variants that occurred in at least 2 samples regardless of the position within the gene.

### 2.5. DNA Methylation

Following bisulfite conversion with the EpiJET Bisulfite Conversion Kit (20 µL of DNA sample containing 200–500 ng of purified genomic DNA; ThermoFisher), the genome-wide methylation levels of the DNA samples were determined using the Infinium MethylationEPIC BeadChip Array (Illumina). The beta values for these arrays were estimated using the minfi package in R [30]. Differentially methylated probes (DMPs) between normal blood (germline DNA) and tRCC, as well as normal kidney (germline DNA) and tRCC (adjusted *p*-value < 0.05), were evaluated and annotated using the ChAMP package in R [31]. To filter the data prior to ChAMP analysis, CpG sites for which beta values across all samples had a maximum value < 0.2 and a minimum value > 0.8 were excluded, as were CpG sites with an absolute maximum difference < 0.2 among all tested samples. The significant DMPs between normal kidney, tRCC, and whole blood samples (Benjamini–Hochberg-adjusted *p*-value < 0.05) were grouped based on K-means clustering with −1 Pearson correlation. All the genes corresponding to hypermethylated probes in tRCC and the genes corresponding to the hypomethylated probes were introduced into ToppFun for functional enrichment analysis [32].

### 2.6. RNA Sequencing

From the 45 tRCC samples passing pathology review for tumor purity metrics (>20% tumor), 36 were selected for RNA analyses, with the great majority having tumor purity of 90–100%. Total RNA (500 ng as determined by Invitrogen Qubit (ThermoFisher)) was poly A selected and reverse transcribed using Illumina’s TruSeq stranded mRNA library preparation kit. Following 12 cycles of PCR amplification, libraries were sequenced on an Illumina HiSeq4000, generating approximately 50 million reads per sample. The RNA sequencing results (unaligned BAM files) of the TCGA RCC patients were downloaded from the cancer.gov web portal. For both the tRCC and TCGA cases, Kallisto (version 0.46.0) [33] was used to pseudo align the reads and assess transcript abundances from FASTQ files. Initially, we constructed an index from the cDNA fasta file sourced from Ensembl (GRCh38.v110). Subsequently, we quantified transcript abundances through the utilization of the Kallisto ‘quant’ function, which processed paired-end read files. The resulting counts and Transcripts Per Million (TPM) values were extracted individually for each sample and then concatenated for further downstream analysis. The TPM counts were transformed to 1-Log2(TPM) counts. Differential gene expression was analyzed using DESeq2 on VST normalized raw counts [34], and genes contributing to the principal components were determined using factoextra [35]. Post-quantification, TPM matrices from both cohorts were merged, and batch effects between the datasets were corrected as much as possible using the ComBat algorithm (sva package in R) using a reduced gene set of the top 7500 genes by ranked expression in both tRCC and tCGA datasets, prior to principal component and correlation analyses.

### 2.7. Fusion Detection

RNAseq fusion detection was performed on the tRCC cases using a custom-developed bioinformatics pipeline in the Molecular and Genomic Pathology Services (MGPS) laboratory. Briefly, paired-end FASTQ files were processed using Fastp to trim adapter sequences, low-quality bases (Q < 20), and filter low-quality reads (read length < 50 bp) [36]. Cleaned FASTQ files were aligned to the human reference genome build GRCh38 using a universal RNA alignment algorithm, STAR aligner v2.7.11, with fusion-sensitive parameters, including minimum split read length, minimum overhang for chimeric junctions, minimum mapped length for spliced mates, and threshold score to rescue chimeric reads [37]. The aligned reads were consumed by two downstream fusion calling algorithms, namely STAR-fusion v1.11 [38] and Arriba [39]. Fusion chimeric reads were detected using a combination of reads spanning the fusion breakpoint and discordant mate pairs. More specifically, filtering criteria included ≥2 split reads and ≥3 discordant pairs (assigned by Arriba as ‘medium/high’ confidence), and in-frame fusions retaining functional domains were prioritized. In addition, fusions flagged as duplicates or with low expression (TPM < 1 in either gene) were excluded. Known fusions were cross-referenced with the Mitelman Database, COSMIC, and TCGA fusions. Novel fusions were evaluated for recurrence in tRCC cohort, retention of oncogenic domains (via Pfam/InterPro), and proximity to regulatory regions in GRCh38. Putative fusion calls were visualized using STAR Fusion Inspector [38] and the Arriba Visualization tool [39]. Putative fusions were manually reviewed by a molecular pathologist to prioritize and identify the driver fusion in the tumor.

### 2.8. Immune Cell Deconvolution

The relative abundance of immune cells in our tRCCs, as well as the TCGA RCC samples, was calculated using the CIBERSORT algorithm from the IOBR/IOBR R package. The immune cell signature matrix from CIBERSORTx, LM22, was used [40]. LM22 consists of 547 genes that accurately distinguish 22 mature human hematopoietic populations, including 7 T-cell types, naïve and memory B cells, plasma cells, NK cells, and myeloid subsets. Wilcoxon Rank-Sum Tests have been performed, comparing the immune cell proportions between tRCC subtypes, as well as between pediatric and adult (TCGA) tRCCs.

### 2.9. Fetal Kidney and Wilms Tumor Expression Atlas

The fetal kidney and Wilms tumor signature matrix were built from single-cell expression data of fetal kidney and Wilms tumors, available via: https://cellxgene.cziscience.com/collections/13d1c580-4b17-4b2e-85c4-75b36917413f (accessed on 17 November 2023) [41].

### 2.10. Survival Analysis

Clinical outcome data were available for 31/41 tRCC patients from the AREN03B2 cohort. Event-free survival (EFS) was defined as the time from diagnostic surgery to the earliest of relapse or progression, second malignancy, or death due to any cause. Overall survival (OS) was defined as time from diagnostic surgery to death due to any cause. Patients not experiencing events of interest were right censored. Kaplan–Meier estimates of EFS and OS were calculated using the survival (version 3.5–7) and survminer R packages (version 0.4.9).

## 3. Results

### 3.1. Patient Demographic and Outcome Data

**Figure 1:**
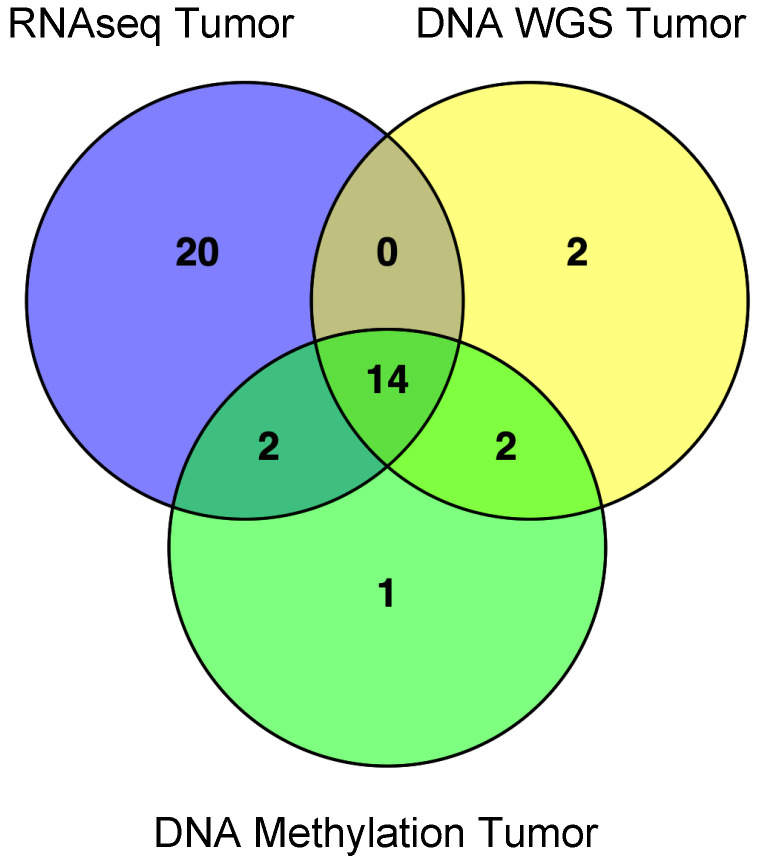
Venn diagram indicating overlaps of the 41 tRCC tumor samples successfully profiled via RNAseq, whole-genome sequencing, and DNA methylation profiling of tumor DNA. Not show are the accompanying non-tumor DNA samples (12 blood, 7 normal kidney) also subjected to DNA methylation profiling.

**Figure 2:**
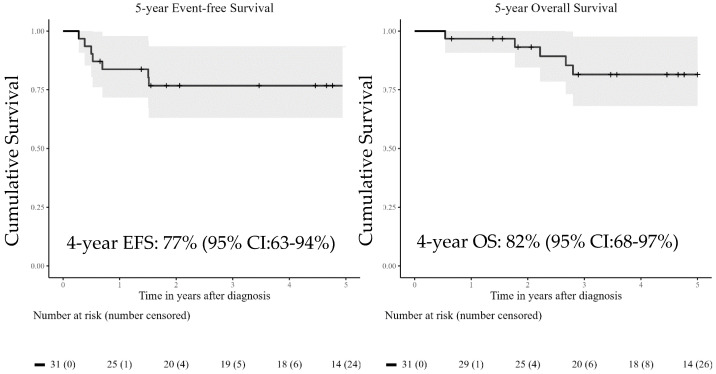
Five year event-free survival (EFS) (A) and five-year overall survival (OS) curves. Reported survival rates are four-year EFS and OS rates.

From the AREN03B2 study, 41 patients were identified with tRCC and sufficient tumor RNA and/or tumor and germline DNA for further molecular analysis. RNAseq data were obtained for 36 patients, whole-genome sequencing data were obtained for 18 patients (including 14 patients with available RNAseq data), and methylation data were obtained for 19 patients (including 14 patients with available RNAseq and WGS data; Figure 1). The basic characteristics of the cohort are described in Table 1. The median age of the patients in our cohort was 11.91 years. Most patients were female, presented with an T3a local stage tumor (tumor invades adrenal gland or perinephric tissues but not beyond Gerota’s fascia), and had no metastases at diagnosis, excluding lymph node metastases. The latter were found in almost half of all patients. Four-year estimated event-free survival was 76.8% (95% Confidence Interval [CI]: 63.0–93.5%), and four-year overall survival was 81.5% (95% CI: 68.1–97.6%) (Figure 2).

### 3.2. Fusion Partners

**Figure 3:**
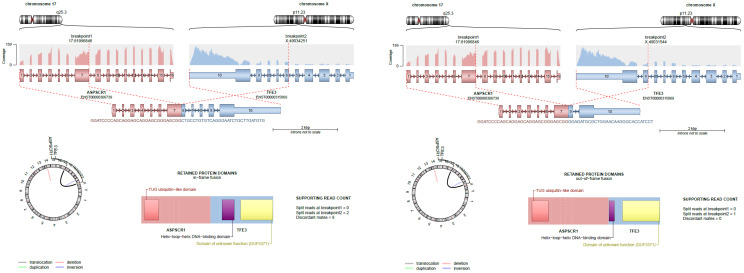
Visualization of the ASPL::TFE3 fusion and reciprocal TFE3::ASPL fusion in the same tRCC sample (PASTEW). Note that although both fusions lead to inclusion of some of the TFE3 DNA binding domain exon, and significantly increased counts of the fused 3’ TFE3 exons, only the exon6 fused transcript leads to an in-frame fusion product.

Initially, the TFE-fusion partner (reported as ‘original annotation’ in Table 2) was determined primarily based on FISH analysis [1]. Detection of TFE-fusion partners was also subsequently assessed during the process of analyzing RNAseq and WGS data from the current study. In 39 out of 41 patients, a TFE3/TFEB fusion could be detected (2 only with STAR-fusion analysis, 4 only with Ariba, and 3 based only on WGS) (Table 2). Fifteen were determined to have an ASPL::TFE3 fusion, and in eight of these, the reciprocal TFE3::ASPL fusion was also detected (example in Figure 3). Eleven patients showed a translocation involving fusion partner PRCC (nine PRCC::TFE3, including four with reciprocal TFE3::PRCC and two patients with only the TFE3::PRCC fusion). Other fusions identified include SFPQ fusions (four; three SFPQ::TFE3 and one TFE3::SFPQ); TFE3::RBM10 fusion (one); MED15::TFE3 and reciprocal TFE3::MED15 fusion (one); and TFEB fusions (three; one TFEB:CLTC, one TFEB:MALAT1, and one unknown). No cases of TFEB amplification, which can occur with or without concurrent TFEB gene rearrangement, were identified [10]. Three samples were negative for any TFE fusion. The original annotation was confirmed in 30 patients (including two cases that were negative for a fusion). A different TFE-fusion partner was detected in three patients, and a previously unknown fusion partner was uncovered in six. In two cases, the original fusion annotation could not be confirmed and currently remains unknown. Although differential exons were included in the original and reciprocal RNA fusion mutants, and in such cases, the fusions showed some differences in gene expression, the difference was not strong enough for further stratification in this study. Hence, the final annotation as presented in Table 2 was used for patient stratification.

### 3.3. Whole-Genome Sequencing

**Figure A1:**
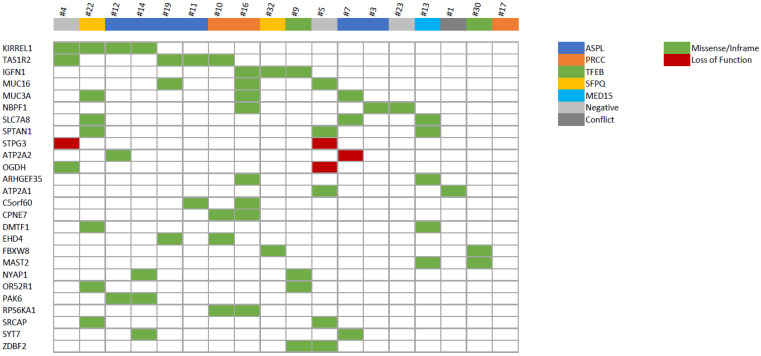
Summary of mutation analysis based on whole-genome sequencing reveals very few recurrent mutations (missense/in-frame and/or loss-of-function mutation in ≥2 samples). Full list of interrogated genes in Supplementary File S1.

**Figure A2:**
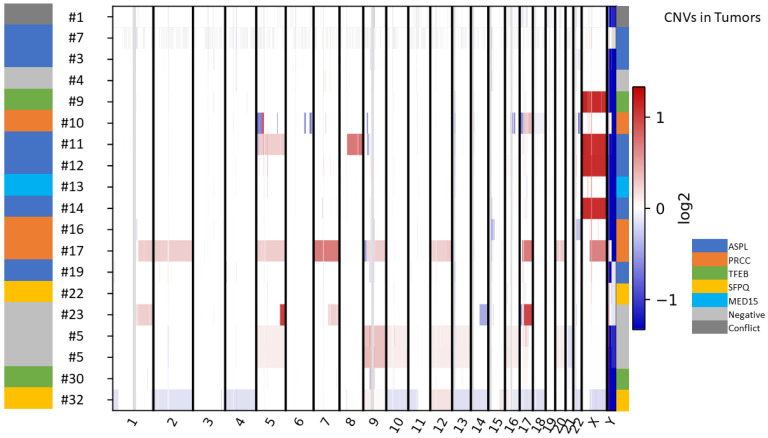
Copy number variations based on WGS analysis reveals gain of 17q in 3 patients.

Whole-genome sequencing data were available for eighteen samples (six ASPL fusions, three PRCC fusions, two SFPQ fusions, two TFEB fusions, one MED15 fusion, three negative samples, and one unknown) Only 26 genes were mutated in at least two patient samples (either missense/in-frame or loss-of-function mutations; Figure A1 in Appendix A and Supplementary Table S1), including mutations in genes that previously been found associated with RCC, such as MUC3A, MUC16, IGFN1, and PAK6 [42,43,44,45]. This may suggest that the TFE3 or TFEB fusions combine with specific mutations to drive tRCC tumorigenesis. One sample that was negative for any gene fusion had a remarkably high number of mutations (188 missense/in-frame and 17 loss-of-function mutations). Copy number variants (CNVs) also were rare, though we identified a 17q gain in 3 out of 18 patients (Figure A2 in Appendix B).

### 3.4. RNA Sequencing

**Figure 4:**
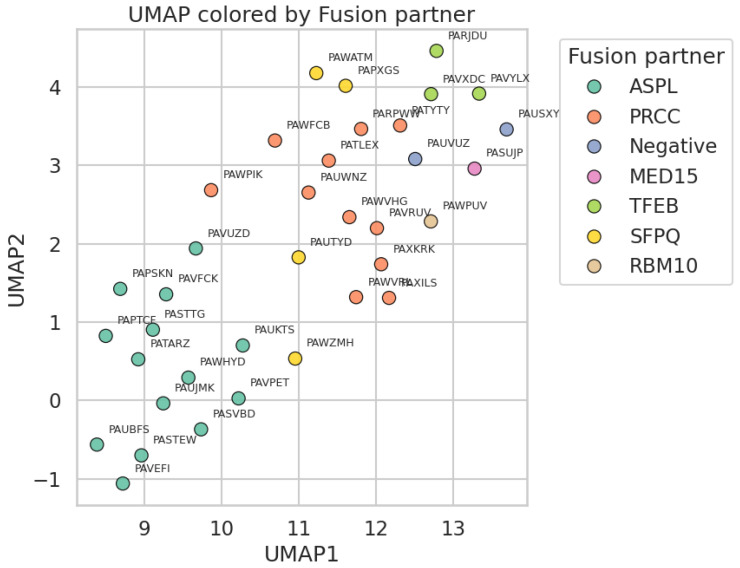
Principal component analysis (PCA) plot of 36 tRCC samples. The plot displays the first two principal components (PC1 and PC2), which, respectively, account for 13.2% and 11.8% of the total variance in the dataset. Each point represents an individual sample, with colors indicating the tRCC subtype based on the fusion partner.

**Figure 5:**
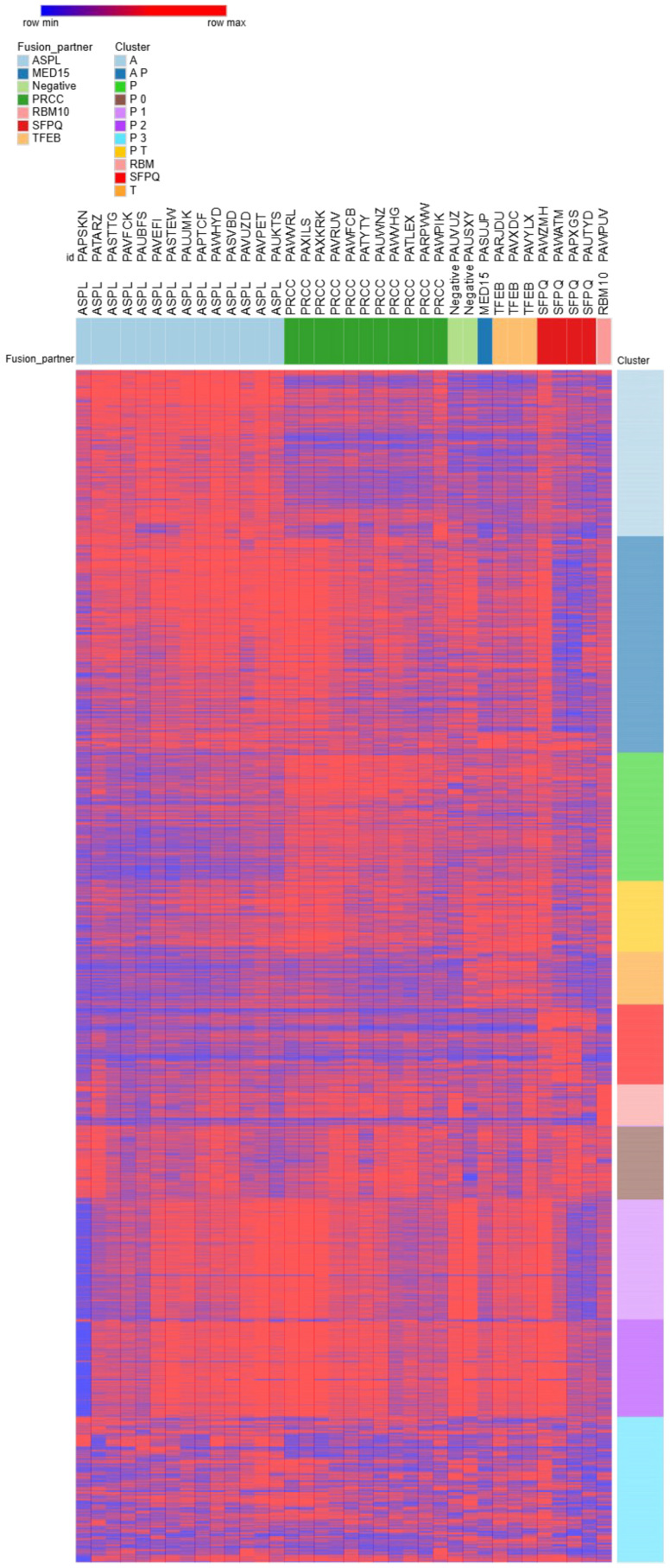
Heatmap of 36 tRCC samples shows distinct transcription patterns per fusion subtype. Each column represents a tumor sample, grouped by its respective fusion subtype, and each row corresponds to a gene. The heatmap displays gene expression levels as log_2_(1 + TPM) counts, with red indicating higher expression and blue indicating lower expression relative to the mean. Hierarchical clustering was applied to the rows (genes) to highlight expression pattern similarities.

RNA sequencing data were generated for 36 samples (14 ASPL fusions, 11 PRCC fusions, 4 SFPQ fusions, 3 TFEB fusions, 1 MED15 fusion, 1 RBM10 fusion, and 2 negative samples). The PCA plot including all 36 samples, labeled by fusion partner, is depicted in Figure 4. The top 500 most differentially expressed genes per tRCC subtype compared to all other subtypes, as well as the top 500 contributing genes for each of the first 10 principal components in PCA, resulted in a list of 6.030 unique genes of interest. Based on supervised clustering, including all differentially expressed genes, another 765 genes that most significantly distinguished between the translocation subtypes were included in the analysis (Figure 5). Each translocation product appeared to drive a strong and specific portion of the transcriptome, mostly related to the different stages of epithelial differentiation.

The heatmap of 36 tRCC samples (Figure 5) revealed a clear separation of the samples into two distinct clusters for PRCC and ASPL, suggesting divergent transcriptome profiles of these two groups. Specifically, we found that PRCC-TFE3 tumors—unlike ASPSCR1 variants—exhibit a unique ‘Epitranscriptomic–Immune’ axis. This includes the specific upregulation of the RNA methyltransferase METTL3 (linked to the splicing function of the PRCC partner) and the macrophage chemoattractants CSF1 and CCL2. We compared the gene expression of the tRCC subtypes to that of fetal kidney and Wilms tumors to correlate the gene expression patterns to stages of (aberrant) embryonal kidney development. From this comparison, we observed that the expression pattern of ASPL::TFE3 tumors resembled that of fetal collecting duct, whereas the PRCC::TFE3 tumors were more similar to fetal proximal tubules. The relative expression of fetal-like (progenitor) epithelial genes and fetal-like (progenitor) mesenchymal genes among the different tRCC subtypes is still actively being studied in our cohort. Notwithstanding, we did observe that players in renal epithelial differentiation that were differentially expressed between all tRCC fusion partners (Supplementary Table S2) were related to different cancer pathways, primarily through Wnt signaling (WNT7B, WNT9B, and CTNNB1) and cyclin-dependent kinase inhibitors (CDKN1A and CDKN2A), which are related to the cell cycle and apoptosis.

### 3.5. DNA Methylation in Combination with Gene Expression

**Figure 6:**
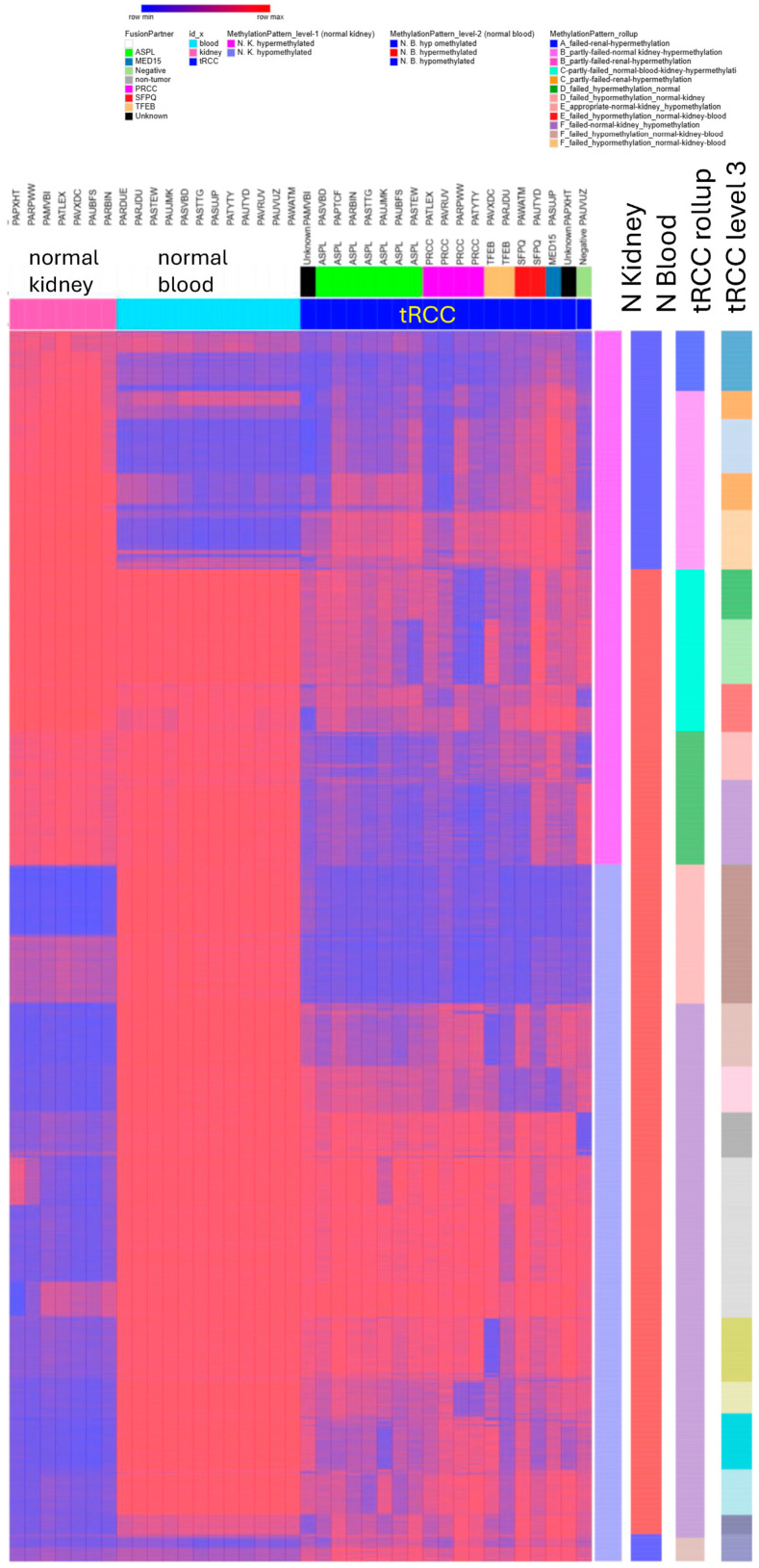
Genome-wide DNA methylation signature of pediatric translocation renal cell carcinoma (tRCC) relative to normal blood and normal kidney.

DNA methylation data were available for nineteen samples (six ASPL fusions, three PRCC fusions, two SFPQ fusions, two TFEB fusions, one MED15 fusion, three negative samples, and two unknown). Upon investigation of the differentially methylated probes (DMPs) between a normal kidney and tRCC (72.9% of these probes were also differentially methylated between normal blood and tRCC), the methylation signature of pediatric tRCC shows less subtype heterogeneity than RNAseq (Figure 5 and Figure 6). Nonetheless, tRCC methylation did show a strong signature of oncogenic transformation with gene sets that were hypomethylated compared to normal kidney, as well as gene sets that were hypermethylated compared to normal kidney.

Heatmap of CpG β-values (0 = unmethylated, 1 = fully methylated) for differentially methylated probes (DMPs) identified by Infinium MethylationEPIC BeadChip profiling (Illumina) of normal blood, normal kidney, and pediatric tRCC tumor samples. Columns represent individual samples; the top annotation bars indicate tissue type and (for tumors) the fusion partner. β-values were estimated using minfi in R [30]. DMP detection and annotation were performed with ChAMP in R [31] for comparisons of tRCC vs normal blood and tRCC vs normal kidney (Benjamini–Hochberg adjusted *p* < 0.05). Prior to DMP calling, CpG sites were filtered to remove low-variance loci (sites with max β < 0.2 or min β > 0.8 across all samples) and loci with limited effect size (max–min β difference < 0.2 across all tested samples). For visualization, values are displayed scaled within each probe (row) from row minimum (blue) to row maximum (red). Significant DMPs were grouped by k-means clustering using 1 − Pearson correlation distance. Right-side tracks summarize probe-wise patterns across sample groups, including mean β-values for normal kidney and normal blood, and the cluster groupings used for the tRCC “roll-up” and “level 3” annotations. Genes corresponding to tumor hypomethylated and hypermethylated probes were used for functional enrichment analysis with ToppFun [32] (see Supplementary Table S3).

Genes hypomethylated compared to normal kidney are strongly enriched for annotations associated with immune cell physiology, cell-mediated immunity, and extracellular matrix organization. In addition, this gene set included cancer-related genes such as MYC, CTNNB1, SHH, FLT3, TGFB1, TGFBR2, and, interestingly, MITF. Genes hypomethylated compared to normal kidney and blood) are enriched for neuronal, epithelial, stromal and mesenchymal to epithelial transition, and tube morphogenesis processes. Similarly, the genes that were hypermethylated (compared to normal kidney) were involved in extracellular matrix organization, neuron development, and signaling through receptor tyrosine kinases, including ERBB, VEGF, Wnt, MAPK, and PI3K-AKT-mtTOR signaling. A subset of the differentially hypomethylated and hypermethylated genes was also found to be differentially expressed among the tRCC subtypes and among the TCGA RCCs and tRCCs when compared to normal kidney samples. The methylation data reveal a critical pediatric-specific biological distinction. Pediatric tRCC is characterized by the profound hypermethylation (silencing) of master nephrogenic progenitor genes (SIX2 and SALL1), which stands in stark contrast to Wilms tumors, where these genes remain active. Conversely, pediatric tRCC exhibits hypomethylation of a distinct neurodevelopmental program (RET, ROBO2, IGF2BP1, EPHA4, and MMP14) that is typically restricted in normal renal development. This suggests that while methylation subtype heterogeneity is lower than in adult RCC, the global epigenetic reprogramming of pediatric tRCC represents a specific ‘identity theft’, rejecting the nephron lineage to access an invasive neural–mesenchymal state. All together, these findings suggest that tRCCs may be driven, in part, by dysregulated epigenetic control.

### 3.6. Shared Potentially Targetable Molecular Pathways

Following ToppFun analysis, we observed that, at the transcriptome level, the tRCC subtypes shared differentially expressed gene sets related to apoptosis, immune regulation, extracellular matrix/cell adhesion, and angiogenesis when compared to normal kidney (based on TCGA data). On a methylome level, we similarly observed dysregulated immunity and extracellular matrix/cell adhesion. Additionally, the methylome revealed dysregulation of genes involved in tyrosine kinase signaling.

**Figure 7:**
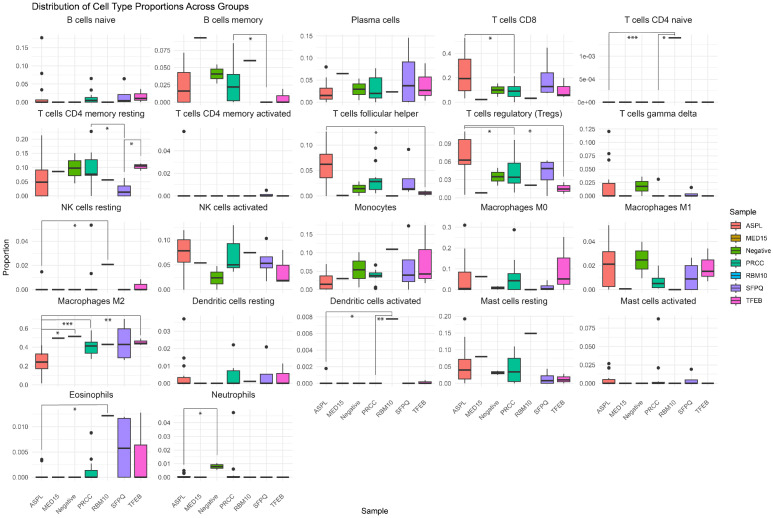
Immune cell deconvolution per pediatric tRCC subtype using CIBERSORTx. *p*-values from Wilcoxon Rank-Sum Test are indicated with asterisks: *: *p* < 0.05, **: *p* < 0.01, and ***: *p* < 0.001.

**Figure 8:**
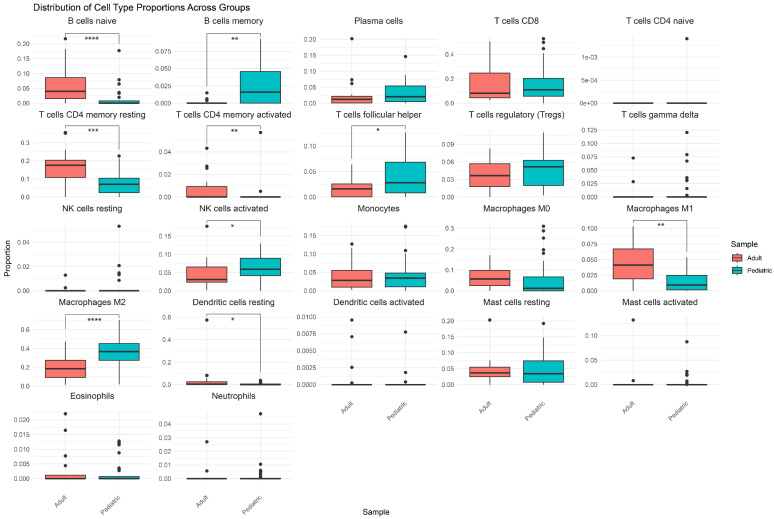
Immune cell deconvolution of TCGA tRCC samples using CIBERSORTx. *p*-values from Wilcoxon Rank-Sum Test are indicated with asterisks: *: *p* < 0.05, **: *p* < 0.01, ***: *p* < 0.001, and ****: *p* < 0.0001.

We performed tumor microenvironment deconvolution to further understand the immune composition of tRCCs. Firstly, analysis confirmed that WT1 expression, typically high in normal glomeruli and Wilms tumors, was essentially absent in our tRCC cohort. This ‘negative signal’ serves as an internal biological control, confirming that our RNA-sequencing data (and subsequent immune deconvolution) reflect the pure tumor-intrinsic profile, free from significant contamination by normal renal elements. Deconvolution of bulk RNAseq data with CIBERSORTx predicted that M2 macrophages and CD8-positive T cells are the immune cell types with the highest relative abundance in the tRCC samples (the full results of CIBERSORTx analysis are reported in Supplementary Table S4). Comparing the immune cell composition between tRCC subtypes, ASPL::TFE3 samples were enriched for CD8-positive T cells, compared to PRCC::TFE3 samples (*p*-value: 0.028674). TFEB and PRCC::TFE3 fusions, as well as samples that were negative for a fusion, contained a larger fraction of M2 macrophages compared to ASPL::TFE3 samples (*p*-values: 0.002941, 0.000677, and 0.01667, respectively). Moreover, tumors with ASPL rearrangements contained a larger fraction of regulatory T cells compared to TFEB fusions (*p*-value: 0.04369) and a larger proportion of follicular helper T cells compared to TFEB and PRCC fusion types (*p*-values: 0.03235 and 0.04423, respectively) (Figure 7). The immune cell compositions of adult tRCC cases included in the TCGA dataset with unknown fusion are presented in Figure 8. Comparing the adult tRCC immune cell composition to our tRCC samples, we observed that the pediatric tRCC samples were composed of relatively more memory B cells (*p*-value: 0.0016) and fewer naïve B cells (*p*-value: 4.4 × 10^−5^). In addition, the pediatric tRCC samples were predicted to have relatively more M2 macrophages (*p*-value: 8.1 × 10^−5^) and fewer M1 macrophages (*p*-value: 0.0025). Finally, the pediatric tRCC contained a larger proportion of activated NK cells (*p*-value: 0.0336) and a smaller proportion of resting and activated CD4 memory T cells (*p*-values: 0.0004 and 0.0019, respectively). These findings are in line with the enrichment of myeloid cell-related genes among the differentially expressed genes between normal kidney samples and pediatric tRCC observed from ToppGene analysis.

The most predominant genes in extracellular matrix organization-related pathways were the metalloproteinases: matrix metalloproteinases (MMPs), a disintegrin and metalloproteases (ADAMs), and a disintegrin and metalloproteinase with thrombospondin motifs (ADAMTSs). The set was also enriched for genes involved in cell motility through mesenchymal-to-epithelial transition (MET) and ERBB signaling (through collagens, integrins, and laminins), as well as focal adhesions and cell junctions, regulated by claudins and cadherins.

**Figure 9:**
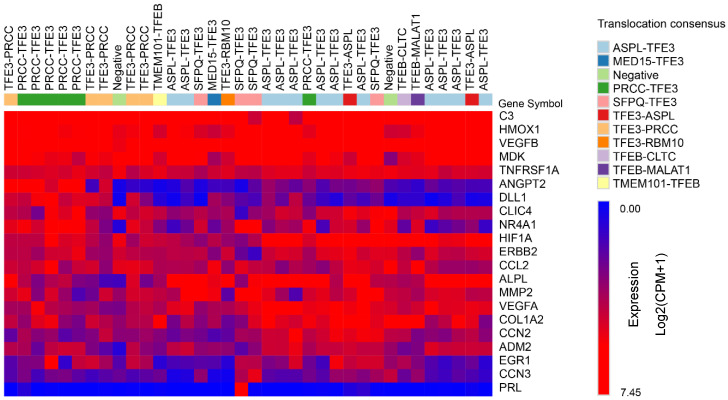
Heatmap of expression levels for angiogenesis drivers shows differential expression of ANGPT2 and VEGFA.

From the 89 genes that were included in the angiogenesis gene set in our cohort, we identified 21 angiogenesis drivers of interest. Expression of these drivers, following unsupervised clustering on the tRCC subtypes, is shown in Figure 9. Both VEGF-A and VEGF-B, targets of commonly used anti-angiogenesis drugs in RCC, are included among the angiogenesis drivers. The expression of VEGF-B is seen across all samples, whereas the expression of VEGF-A is lower in tumors with a TFE3-PRCC fusion. In contrast, tumors with PRCC-fusion uniquely showed high expression of ANGPT2. The expression pattern of VEGF-A was highly like that seen for HIF1A.

Finally, pathway analysis on the signal transduction gene set through ToppGene showed shared enrichment for signaling by receptor tyrosine kinases across all tRCCs and, more specifically, the PI3K-AKT-mtTOR and MAPK pathways.

### 3.7. The Place of Pediatric tRCC Among Adult RCC

To accurately position pediatric tRCC within the broader landscape of renal neoplasia, we integrated our cohort with the complete adult TCGA renal cell carcinoma datasets (KIRC, KIRP, and KICH). Recognizing that technical variation between independent datasets can obscure true biological signals, we applied the ComBat algorithm to correct for batch effects, utilizing a rigorously filtered set of the top 7500 most variable genes (log2(TPM + 1) ≥ 2.0).

**Figure 10:**
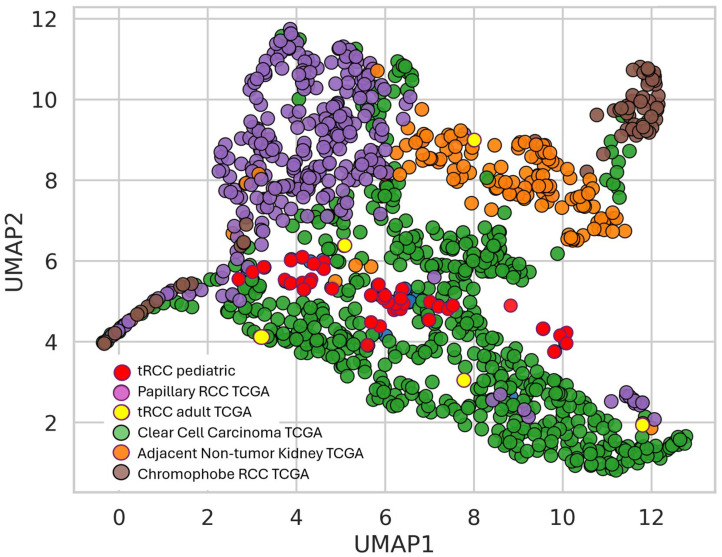
TPM counts of TCGA and pediatric tRCC samples; UMAP dimensionality reduction analysis of transcriptomic profiles comparing pediatric tRCC to adult renal tumors (TCGA). Uniform Manifold Approximation and Projection (UMAP) visualization assessing the global transcriptional relationship between the pediatric tRCC cohort (*n* = 36; red) and adult renal tumors from TCGA, including Clear Cell RCC (green), Papillary RCC (purple), Chromophobe RCC (brown), adult tRCC (yellow), and adjacent normal kidney tissue (orange). To control for technical batch effects, raw counts were reprocessed using an identical Kallisto pipeline and corrected using the ComBat algorithm for the top Fig genes (log2(TPM + 1) ≥ 2.0). The plot reveals that pediatric tRCC samples form a distinct, tightly grouped molecular cluster situated in a transcriptional niche intermediate between adult Clear Cell and Papillary RCCs, widely separated from Chromophobe RCC and normal kidney samples.

Dimensionality reduction via UMAP revealed a striking biological architecture (Figure 10). Unlike prior analyses that may have suggested a predominant overlap with a single adult subtype, the batch-corrected landscape demonstrates that pediatric tRCC constitutes a distinct molecular entity. The pediatric tRCC samples (red) clustered tightly together, confirming high intra-cohort homogeneity. Crucially, they occupied a unique transcriptional niche situated at the interface of adult Clear Cell RCC (green) and Papillary RCC (purple), sharing transcriptomic features with both lineages but forming a separate cluster that does not fully recapitulate either adult profile. This contrasts sharply with adult Chromophobe RCC and normal kidney tissues, from which pediatric tRCCs were widely separated. Interestingly, the few adult tRCC cases identified within the TCGA (yellow) showed greater heterogeneity; while some clustered alongside the pediatric cases, others dispersed into the adult Papillary RCC cluster, potentially reflecting age-dependent biological divergence even within the translocation-positive category.

**Figure 11:**
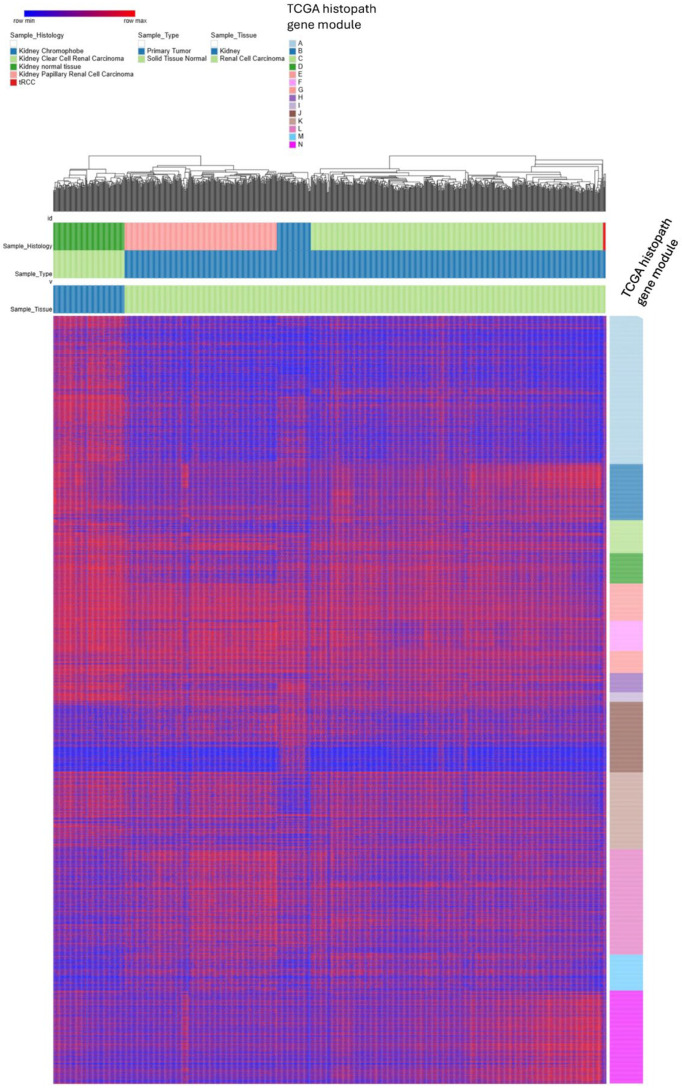
Hierarchical clustering heatmap of signature gene expression modules defining pediatric tRCC relative to adult TCGA renal tumors. This heatmap displays the expression profiles of highly variable genes organized into distinct transcriptional modules (rows) across the combined cohort of pediatric tRCC (*n* = 36) and adult TCGA renal tumors (Clear Cell RCC, Papillary RCC, Chromophobe RCC, and normal kidney). Columns represent individual samples color coded by histology. The rows are grouped into functional gene signature modules identified by differential expression patterns: (1) Normal Kidney-Specific Modules (N1 and N2): genes highly expressed in normal tissue but universally downregulated in all tumor subtypes (including tRCC); (2) Chromophobe-Specific Module (T Chrom high): a distinct signature defining Chromophobe RCC, which is notably absent in pediatric tRCC; (3) Papillary-Specific Module (T high Pap 2): genes enriched in Papillary RCC, showing partial/variable expression in the pediatric tRCC cohort; (4) Clear Cell/Pan-RCC Module (N + T Clear 1): a signature strongly shared between adult Clear Cell RCC and pediatric tRCC, likely reflecting common angiogenic and metabolic dysregulation (HIF pathway); (5) Shared Tumor Suppression Modules (N + T Chrom/Pap/Clear down): gene sets that are universally downregulated across specific tumor lineages compared to normal kidney. The analysis confirms that pediatric tRCC possesses a ‘chimeric’ transcriptome: it is defined by the absence of normal/chromophobe signatures, the retention of the Clear Cell/Pan-RCC program, and specific overlaps with the papillary lineage (see Supplementary Table S5).

To deconstruct the molecular architecture placing pediatric tRCC between adult Clear Cell and Papillary RCCs, we performed hierarchical clustering to identify gene signatures specific to each histological subtype (Figure 11). This analysis revealed that the transcriptome of pediatric tRCC is foundationally ‘modular’, selectively retaining and rejecting specific adult renal programs.

Firstly, pediatric tRCC samples displayed a profound ‘negative signature’ characterized by the complete absence of gene modules defining normal adult kidney and Chromophobe RCC. This confirms the UMAP finding that tRCC is biologically distant from the chromophobe lineage and suggests a loss of mature tubular metabolic functions retained by normal tissue.

Secondly, we identified a shared ‘Pan-RCC/Clear Cell’ signature (Cluster X) strongly expressed in both pediatric tRCC and adult Clear Cell RCC (KIRC), likely representing shared angiogenic or stromal-remodeling pathways driven by VHL-like pseudohypoxia (HIF-pathway activation).

Thirdly, pediatric tRCCs exhibited partial expression of the Papillary RCC signature, aligning with their intermediate position in the UMAP space. However, the most striking feature was a distinct tRCC-Intrinsic Signature (Cluster Y) that was highly expressed in our pediatric cohort but largely absent in adult KIRC, KIRP, and KICH samples. This module likely contains the direct targets of the TFE3 fusion proteins, specifically the Coordinated Lysosomal Expression and Regulation (CLEAR) network genes, defining the unique biological identity of translocation RCC beyond simple resemblance to adult subtypes [46,47]. This validates recent findings linking TFEB/TFE3 fusions to constitutive activation of lysosomal biogenesis and cystogenesis [48]. This module likely contains the direct targets of the TFE3 fusion proteins (e.g., the CLEAR network/lysosomal genes), defining the unique biological identity of translocation RCC beyond simple resemblance to adult subtypes [49].

## 4. Discussion

tRCC represents the largest sub-type of pRCC and is composed of a heterogeneous group of cancers, with a large percent unclassifiable by current techniques and some challenging to distinguish between other forms of RCC based on histology and immunohistochemistry alone. Based on RNAseq and WGS, we were able to determine the translocation type in 39/41 tRCC samples. Similarly, Lee et al. were unable to detect a fusion on the transcriptome level in 4 out of 19 tRCC samples that were TFE3 positive on immunohistochemistry [50]. It has previously been suggested that TFE3 staining can result in false-positive tRCC diagnoses [51]. Gene mutation analyses did not aid in the further distinction between tRCC subtypes, as we identified no recurrent mutations, confirming that the mutational burden in (pediatric) tRCC is low [8,52].

In general, copy number variations (CNVs) that are commonly identified in tRCC include 9p loss and 17q gain [8,53]. Whole-genome analysis, applied to our pediatric tRCC, herein identified 17q gain as a recurrent CNV in 3/18 patients. Two of these patients had a tumor with a PRCC::TFE3 fusion, and one tumor was negative for any fusion. Such diagnostics, in addition to PAX8 immunohistochemistry, may aid in distinguishing tRCC from renal PEComa, which do not typically contain these CNVs [53]. Furthermore, tRCCs with copy number aberrations were associated with poor overall survival in the study by Marcon et al. [53]; however, none of the pediatric patients (*n* = 3) included in that study showed loss of 9p or gain of 17q. As such, it remains unclear if these CNVs have prognostic value specifically in pediatric tRCC patients.

Transcriptomic and genomic profiling applied to other common renal tumors of childhood (Wilms tumor, rhabdoid tumor, and clear cell sarcoma of the kidney) have yielded a wealth of information [54,55,56,57,58]. Applied to adult RCC, such efforts have identified a 7-gene predictor classifying Papillary RCC subtypes into two subgroups with therapeutic and prognostic impact [59]. Transcriptome analysis in our cohort did reveal subtype differences, with each translocation product driving a strong and specific portion of the transcriptome, predominantly related to epithelial differentiation.

In contrast to the transcriptome analysis, the methylation signature in our cohort of pediatric tRCC showed little subtype heterogeneity. Hypomethylation of CpG islands in genes generally results in the upregulation of gene expression, whereas hypermethylation suppresses gene expression [60]. Detailed interrogation of the differentially methylated loci revealed a striking dichotomy representing a ‘switch’ in lineage identity. We observed profound hypermethylation (silencing) of the core transcriptional machinery required for kidney development and function. This included the master nephrogenic stem cell factors SIX2 and SALL1, as well as critical regulators of tubular differentiation such as PAX2, PAX8, HNF1A, and GATA3. Furthermore, genes defining mature renal physiology, including the water channel AQP2, uromodulin (UMOD), and solute carriers (SLC12A1 and SLC22A1), were consistently hypermethylated, confirming a loss of functional renal differentiation. Conversely, the hypomethylated (potentially active) gene set was dominated by an aberrant neuro-mesenchymal program. This included the ‘oncofetal’ RNA-binding protein IGF2BP1, a potent driver of metastasis usually silenced after birth. We also identified robust hypomethylation of axonal guidance genes that are often co-opted for tumor invasion, specifically RET, ROBO2, and EPHA4. This suggests that tRCC does not arise from a simple arrest of the nephrogenic rest but rather from reprogramming where the tumor actively represses its renal identity to access an invasive neural state. Data suggest that whereas the embryonic pluripotent cells are guided to kidney differentiation in normal kidney development, the disrupted differentiation in tRCC appears to result in neuro-like differentiation. Interestingly, it has previously been established that the default fate of embryonic pluripotent cells is neural [61]. In addition, gene sets that failed to correctly methylate in tRCC were enriched for extracellular matrix organization, cell adhesion, and immunity. Similar gene sets were enriched for in the shared transcriptome signature among tRCC subtypes, in addition to negative regulation of apoptosis and angiogenesis. In one study by Malouf et al., adult tRCC methylation profiling demonstrated two clusters, the first of which included Clear Cell RCC, Papillary RCC, mucinous and spindle cell RCC, along with tRCC; cluster 2 included Oncocytic and Chromophobe RCC. Cluster 1 demonstrated more hypermethylation than cluster 2 and a convergence on Polycomb targets [62]. These processes may thus present potential therapeutic targets.

Pathway analysis across all tRCC subtypes identified pathways related to extracellular matrix organization, and specifically, metalloproteinases emerged as interesting targets for tRCC therapy. Various MMPs have been found to be overexpressed in adult RCC, and the expression of MMP-2, MMP-7, and MMP-9, specifically, appears to correlate with advanced RCC and poor outcomes [63]. Inhibitors of MMPs have been found to effectively target cancer cells [64]. Moreover, melatonin has been observed to reduce metastases formation in RCC mouse models through inhibition of MMP-9 (amongst others) [65]. In one published RCC cell line, silencing of NFKB was shown to inhibit colony formation, cell migration, and invasion through downregulation of MMP-9 [66]. Beyond angiogenesis, our analysis identifies a unique ‘Epitranscriptomic–Immune’ axis specific to the PRCC-TFE3 fusion subset. Unlike ASPSCR1-fusions, the PRCC-TFE3 tumors display a distinct upregulation of the m6A RNA methyltransferase METTL3 and the thiol methyltransferases METTL7A/B. Given that the wild-type PRCC protein functions within the nuclear speckle to regulate splicing [67], it is possible that the PRCC-TFE3 fusion may aberrantly recruit METTL3 to fusion-target transcripts, creating a unique splicing–methylation dependency not seen in other tRCCs. Furthermore, this subset is significantly enriched for the myeloid chemoattractants CSF1 and CCL2, which correlates with the increased M2 macrophage infiltration we observed in these samples. Collectively, these data suggest that PRCC-TFE3 tumors rely on a distinct survival mechanism combining ‘thiol shielding’ (METTL7B) and immune-suppressive macrophage recruitment, potentially indicating utility for CSF1R inhibitors or metabolic targeting in this specific subgroup [68].

Immune cell deconvolution analyses performed on pediatric tRCC tumors presented herein indicate that tRCCs have a high relative abundance of M2 macrophages, and ASPL::TFE3 samples were enriched for CD8-positive T cells. Compared to adult RCC, pediatric tRCC samples were composed of relatively more M2 macrophages (except for the ASPL::TFE3 subtype). M2 macrophages have previously been found to be significantly increased in renal cell carcinoma and to promote the migration and invasion of RCC in adult cell lines [69,70]. He et al. suggested that M2 macrophages support growth and metastases formation by inhibiting autophagy through Bcl-2 [70]. Moreover, M2 macrophages have been proposed to enhance angiogenesis through VEGF secretion, as well as influence the potential of cell infiltration by targeting the extracellular matrix through secretion of MMPs [71]. Finally, M2 macrophages in Clear Cell RCC are thought to promote epithelial-to-mesenchymal transition by promoting the AKT pathway [72]. While of interest in both adult Clear Cell and non-Clear Cell RCCs [73,74,75], more recent evidence suggests that the efficacy of dual anti-CTLA4/PD1 therapy is limited for adult (metastatic) tRCC specifically [76,77]. The role for immune checkpoint inhibition in the treatment of pediatric tRCC is still actively being studied.

Tyrosine kinase signaling was also enriched among the tRCC subtypes in our study, and signaling was shown to be dysregulated through methylation. More specifically, the PI3K-AKT and MAPK pathways appeared to be interesting targets [78]. The PI3K-AKT-mTOR pathway regulates cell growth, differentiation, migration, survival, metabolism, and angiogenesis. Mechanistically, transcriptional activation of RagD GTPase by TFE3 fusions has been shown to result in constitutive mTORC1 hyperactivation, bypassing normal nutrient sensing [79]. Both pathways have previously been implicated in RCC. It has been suggested that insensitivity to mTOR inhibition results from mTOR-induced cell death escape via MAPK or compensatory TFEB regulation [80,81,82,83,84]. However, in a single-center retrospective pediatric tRCC cohort, mTOR inhibition was not effective [85]. It has been suggested that insensitivity to mTOR inhibition results from mTOR-induced cell death escape via MAPK, and hence, combination therapy inhibiting both mTOR and MAPK may be a potential option to assess [84]. Chen et al. reported on TFE3-mediated upregulation of GSTP1 expression, which was found to drive tumor progression through JNK signaling, suggesting that targeting this pathway may be a potential therapeutic strategy [86].

The mechanisms of apoptosis regulation in kidney cancer have previously been described by Ganini et al. [87]. In the presented pediatric tRCC cohort, the expressions of BAX, BCL-2A1, and HIF1A were significantly different between tRCCs and normal kidneys. BAX was highly expressed across all tRCC subtypes, whereas the BCL-2 family member BCL-2A1 was specifically expressed in PRCC-TFE3 tumors. In contrast, HIF1A was expressed only in ASPL-TFE3 tumors.

Moreover, VEGF-A and VEGF-B were both among the angiogenesis drivers expressed among our tRCC cohort. Interestingly, whereas the expression of VEGF-B was equal across all samples, the expression of VEGF-A was lower in samples with a PRCC-fusion. In contrast, ANGPT-2 expression was limited to specifically this tRCC subtype. Numerous antiangiogenic agents are FDA approved for adult RCC. Whether selective targeting of VEGF-A vs. ANGPT-2 or alternative angiogenic targets will impact patients with differing tRCC subtypes remains uncertain.

Finally, the literature indicates that regulation of mitochondrial metabolism plays a role in tRCC. Li et al. and Lee et al. reported that TFE3 fusions drive oxidative phosphorylation in adult tRCC (cell lines) [51,88]. In line with these findings, Bakouny et al. found that the upregulated genes in tRCC were related to mTORC1 signaling, sensing of reactive oxygen species, and the response to oxidative stress [8,89]. The proteogenomic characterization study by Qu et al. also indicated dysregulated metabolism in tRCC, and the authors suggested mTOR signaling as a therapeutic target [23]. Li et al. additionally showed that stabilizing HIF-1α, which induced a metabolic shift away from oxidative phosphorylation, may be a therapeutic strategy in these tRCC [88]. In our pediatric tRCCs, this may be relevant especially in PRCC-TFE3 subtypes, in which HIF-1α expression was low.

## 5. Conclusions

In conclusion, we have demonstrated that our pediatric tRCCs had a low mutation burden, while the fusion product generally drove a strong and specific portion of the transcriptome. Specifically, PRCC and ASPL tRCC subtypes showed distinct gene expression patterns. Even though the methylation signature of pediatric tRCC showed less subtype heterogeneity, the data suggest that tRCCs may be driven, in part, by dysregulated epigenetic control. The changes in gene expression and methylation predominantly related to pathways involved in apoptosis, immune regulation, extracellular matrix/cell adhesion, tyrosine kinase signaling, and angiogenesis, thereby providing potential (subtype specific) targets for therapy in pediatric tRCC for further study.

## Figures and Tables

**Table 1:** Patient characteristics.

Characteristics	Number of Patients	%
Total	41	100
Median age (years)	11.91	
Sex		
Female	28	68.3
Male	13	31.7
Race		
Black/African American	16	39.0
White	20	48.8
Asian	1	2.4
Native Hawaiian or other Pacific Islander	1	2.4
Unknown	3	7.3
Local tumor stage		
T1	12	29.3
T2	2	4.9
T3a	19	46.3
T4	1	2.4
Tx	2	4.9
Unknown	3	7.3
Lymph node status		
Positive	19	46.3
Negative	10	24.4
Unknown	12	29.3
Metastases at diagnosis		
Yes	4	9.8
No	34	82.9
Indeterminable	3	7.3

**Table 2:** Samples.

Sample	Germline DNA (Sample Type)	Tumor DNA	Tumor RNA	Original Annotation	Final Annotation	Translocation
1	Yes (Kidney)	Yes	No	ASPL	Unknown	Unknown
2	Yes (Kidney)	Yes *	No	PRCC	Unknown	Unknown
3	Yes (Kidney)	Yes	No	ASPL	ASPL	ASPL::TFE3
4	Yes (Blood)	Yes	No	Unknown	Negative	Negative
5	Yes (Blood)	Yes	No	Negative	Unknown	Unknown
6	No	No	Yes	ASPL	ASPL	ASPL::TFE3
7	No	Yes	Yes	PRCC	ASPL	ASPL::TFE3
8	No	No	Yes	Unknown	SFPQ	SFPQ::TFE3
9	Yes (Blood)	Yes	Yes	TFEB	TFEB	CLTC::TFEB
10	Yes (Kidney)	Yes	Yes	PRCC	PRCC	PRCC::TFE3
11	Yes (Blood)	Yes	Yes	ASPL	ASPL	ASPL::TFE3
12	Yes (Blood)	Yes	Yes	ASPL	ASPL	ASPL::TFE3::ASPL
13	Yes (Blood)	Yes	Yes	Unknown	MED15	MED15::TFE3::MED15
14	Yes (Blood)	Yes	Yes	ASPL	ASPL	ASPL::TFE3::ASPL
15	No	No	Yes	ASPL	ASPL	ASPL::TFE3::ASPL
16	Yes (Kidney)	Yes	Yes	PRCC	PRCC	TFE3::PRCC
17	Yes (Blood)	Yes	Yes	PRCC	PRCC	PRCC::TFE3::PRCC
18	Yes (Kidney)	Yes	Yes	ASPL	ASPL	ASPL::TFE3
19	Yes (Blood)	Yes	Yes	Unknown	ASPL	ASPL::TFE3::ASPL
20	No	No	Yes	ASPL	ASPL	ASPL::TFE3::ASPL
21	No	No	Yes	Negative	Negative	Negative
22	Yes (Blood)	Yes	Yes	Unknown	SFPQ	TFE3::SFPQ
23	Yes (Blood) **	Yes	Yes	Negative	Negative	Negative
24	No	No	Yes	PRCC	PRCC	TFE3::PRCC
25	No	No	Yes	ASPL	ASPL	ASPL::TFE3
26	No	No	Yes	ASPL	ASPL	ASPL::TFE3::ASPL
27	No	No	Yes	ASPL	ASPL	ASPL::TFE3::ASPL
28	No	No	Yes	PRCC	PRCC	PRCC::TFE3
29	No	No	Yes	ASPL	ASPL	ASPL::TFE3
30	Yes (Kidney)	Yes	Yes	TFEB	TFEB	MALAT1::TFEB
31	No	No	Yes	Negative	TFEB	X::TFEB
32	Yes (Blood)	Yes	Yes	SFPQ	SFPQ	SFPQ::TFE3
33	No	No	Yes	PRCC	PRCC	PRCC::TFE3
34	No	No	Yes	ASPL	ASPL	ASPL::TFE3::ASPL
35	No	No	Yes	PRCC	PRCC	PRCC::TFE3::PRCC
36	No	Nlo	Yes	Negative	RBM10	TFE3::RBM10
37	No	No	Yes	PRCC	PRCC	PRCC::TFE3::PRCC
38	No	No	Yes	PRCC	PRCC	PRCC::TFE3
39	No	No	Yes	Unknown	SFPQ	SFPQ::TFE3
40	No	No	Yes	PRCC	PRCC	PRCC::TFE3::PRCC
41	No	No	Yes	PRCC	PRCC	PRCC::TFE3

* Only DNA methylation data available; ** bloodrawn at end of therapy.

## Data Availability

The data presented in this study are available on request from the corresponding author. The data are not publicly available due to privacy restrictions.

## Supplementary Materials

The following supporting information can be downloaded at: https://www.mdpi.com/article/10.3390/biomedicines14050955/s1, Table S1: Whole Genome Sequencing Table: The table lists all gene fusions found through the combined analyses of whole genome sequencing and RNAseq fusion gene detection algorithms as described in the methods sections; Table S2: Transcriptome that correlates with fusion genes—The table lists all genes shown in the heatmap, their module designations, as well as all of the metadata attributes of each patient from which that sample was derived; Table S3: Differentially methylated genes defining the epigenetic landscape of pediatric translocation renal cell carcinoma (tRCC). Gene-level summary of loci showing significant differential methylation (BH-FDR < 0.05) between pediatric tRCC tumors and normal kidney tissue profiled on the Infinium MethylationEPIC array. Genes are grouped by the directionality of probe-level change in tumors: Hypomethylated (blue; decreased β in tRCC, consistent with open chromatin/potential reactivation), Hypermethylated (pink; increased β in tRCC, consistent with transcriptional repression), and Mixed/Complex (yellow; loci containing both hyper- and hypomethylated probes). Key recurring patterns include: (A) Hypomethylated (“reactivated”) genes enriched for neurodevelopment/axon-guidance and oncogenic/invasion programs (e.g., RET, ROBO2, EPHA4, IGF2BP1, MMP14, CD44, and WNT5A); (B) Hypermethylated (“silenced”) genes enriched for nephrogenic progenitor and renal lineage transcription factors and mature tubular transport programs (e.g., SIX2, SALL1, PAX2, PAX8, HNF1A, AQP2, SLC12A1, and UMOD), consistent with suppression of kidney identity; and (C) Complex regulation, including developmental regulators such as WT1 and WNT4 with heterogeneous probe-level methylation patterns consistent with a “locked” epigenetic state distinct from Wilms tumor. Gene sets derived from the hypomethylated and hypermethylated categories were used for downstream enrichment analysis (ToppFun [32]); Table S4: Cibersort analysis of the relative proportions of each of the immune cell types in each of the tRCC samples. Estimated immune cell composition of pediatric tRCC tumors. Relative fractions per sample were inferred using CIBERSORT (bulk RNA-seq, LM22 matrix). P-value (permutation-based) and Pearson correlation indicate deconvolution confidence. Values represent proportions within the immune compartment; Table S5: Gene membership of transcriptional signature modules correlating with renal tumor histology. TCGA histology-associated gene modules used to co-cluster pediatric tRCC with the adult TCGA renal cancer expression atlas are listed as transcriptional modules visualized in Figure 11. The heatmap shows both the relatively and consistent and subtype patterns of the different histological subtype of renal carcinomas and represent the subset of the 7500 genes that have the highest significance for histological classification. Pediatric tRCC and TCGA expression matrices were merged and adjusted for cross-cohort technical effects using ComBat, and the resulting integrated matrix was used for unsupervised clustering. Columns represent individual samples and are annotated (top) by histologic diagnosis (chromophobe RCC, clear cell RCC, papillary RCC, normal kidney, and pediatric tRCC), specimen type (primary tumor vs solid tissue normal), and tissue class (kidney vs RCC). Rows represent genes grouped into the labeled TCGA Renal Cancer Atlas gene modules (A–N)—the co-expression modules most significantly associated with histological tumor type—with module membership indicated by the row-side color bar. Expression values are displayed scaled within each gene (row) (blue = lower relative expression; red = higher relative expression for that gene across samples), and the column dendrogram shows unsupervised hierarchical clustering based on these module genes.

## Abbreviations

The following abbreviations are used in this manuscript:

tRCC	Translocation morphology renal cell carcinoma
WGS	Whole-genome sequencing
RCC	Renal cell carcinoma
pRCC	Pediatric renal cell carcinoma
TFE3	Transcription factor E3
TFEB	Transcription factor B
FISH	Fluorescence in situ hybridization
LOH	Loss of heterozygosity
SNP	Single-nucleotide polymorphism
IRB	Institutional review board
TCGA	The Cancer Genome Atlas
FFPE	Formalin-fixed, paraffin-embedded
DMP	Differentially methylated probe
TPM	Transcripts per million
EFS	Event-free survival
OS	Overall survival
CNV	Copy number variant
PCA	Principal component analysis
MMPs	Matrix metalloproteinases
ADAMs	A disintegrin and metalloproteases
ADAMTSs	A disintegrin and metalloproteinase with thrombospondin motifs
